# Use of first trimester hemoglobin albumin lymphocyte platelet score and fibrinogen to albumin ratio in the prediction of pre-eclampsia

**DOI:** 10.1097/MD.0000000000043423

**Published:** 2025-07-25

**Authors:** Murat Polat, Mehmet Alican Sapmaz, Sait Erbey, Aziz Kindan, Muhammed Said Ruzgar, İnci Kahyaoglu

**Affiliations:** a Department of Obstetrics and Gynecology, Ankara Etlik City Hospital, Ankara, Turkey; b Department of Perinatalogy, Ankara Etlik City Hospital, Ankara, Turkey.

**Keywords:** albumin, fibrinogen, hemoglobin, lymphocyte, platelet, pre-eclampsia

## Abstract

Pre-eclampsia (PE) is a severe pregnancy complication associated with increased maternal and fetal morbidity and mortality. In this retrospective study, we aimed to evaluate the predictive value of hemoglobin–albumin–lymphocyte–platelet score and fibrinogen/albumin ratio (FAR) for early diagnosis of PE. Medical records of 252 pregnant women categorized as severe pre-eclampsia, non-severe pre-eclampsia and control groups were analyzed. Biomarker levels including hemoglobin, albumin, lymphocytes, fibrinogen, creatinine, alanine transaminase, aspartate aminotransferase, and platelets were analyzed throughout the first trimester. The results showed that FAR was significantly higher in PE groups compared to controls and correlated with disease severity (area under curve = 0.65, sensitivity = 59%, specificity = 66%, *P* < .001). Albumin levels were moderately predictive for PE (area under curve = 0.70, sensitivity = 66%, specificity = 71%, *P* < .001). In contrast, the hemoglobin–albumin–lymphocyte–platelet score did not differ significantly between groups, indicating limited utility for early prediction. Elevated FAR is thought to result from increased fibrinogen levels due to inflammatory and procoagulant mechanisms, whereas decreased albumin levels reflect systemic inflammation associated with PE. Although FAR and albumin show potential as early biomarkers for PE, their diagnostic power alone is moderate. The findings suggest that these parameters may be more effective when combined with established markers such as placental growth factor and soluble fms-like tyrosine kinase-1. The study underlines the need for larger prospective studies to confirm these findings and explore additional biomarkers for improved PE prediction.

## Graphical abstract

**Figure:**
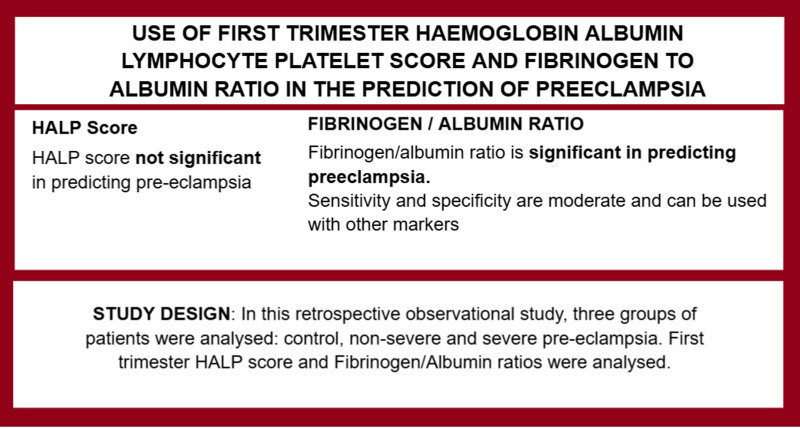

## 1. Introduction

Pre-eclampsia (PE) is a disease that complicates approximately 5% of pregnancies and can lead to serious complications for both mother and fetus.^[1]^ According to the definition of the International Society for the Study of Hypertension in Pregnancy, PE is an obstetric condition in which systolic blood pressure (BP) ≥ 140 mm Hg and/or diastolic BP ≥ 90 mm Hg, starting after the 20th week of gestation are accompanied by proteinuria, uteroplacental dysfunction and end organ damage.^[2]^ The American College of Obstetricians and Gynecologists classifies PE as severe pre-eclampsia (SP) and non-severe pre-eclampsia (NSP).^[3]^ PE is associated with serious complications including placental insufficiency, preterm labor, intrauterine growth retardation, maternal and infant mortality.^[4]^ Therefore, prediction of PE in early gestational weeks is important in terms of early initiation of acetylsalicylic acid use for prophylaxis and prevention of PE.

Various tests have been investigated for the prediction of PE. Among these tests, placental-derived biomarkers such as placental growth factor (PlGF) and soluble fms-like tyrosine kinase-1 (sFlt-1) are prominent and play an important role in the diagnosis and prognosis of PE.^[5–7]^ In recent years, various biomarkers related to inflammation and nutritional status have also been the subject of research and in this context, hemoglobin (Hb), albumin (Alb), lymphocyte (Lym), and platelets’ (Plt) hemoglobin–albumin–lymphocyte–platelet (HALP) score and fibrinogen/albumin ratio (FAR) stand out as 2 remarkable parameters in the diagnosis of PE. HALP score is a haematological index including Hb, Alb, Lym, and Plt values. The formula of this score is as follows: Hb × Alb × Lym/Plt. HALP score was first defined as a prognosis indicator in oncological diseases, and then its use in different clinical conditions was investigated.^[8]^ Dal et al showed that the HALP score was lower in PE patients compared with healthy controls in a study performed in 2024.^[9]^

The FAR is a biochemical index reflects the inflammation and coagulation. This ratio is calculated by dividing the patient’s serum fibrinogen (Fib) level by the serum Alb level. Fib is a protein involved in the blood clotting process and is a positive acute phase reactant that increases during inflammatory response. Alb, on the other hand, is a negative acute phase reactant that reflects nutritional status and general health status and whose serum level decreases in inflammation. Ren et al, it was reported that FAR values increased in PE patients and this increase was positively correlated with the severity of the disease.^[10]^

As a result of abnormal placentation in the first trimester in PE patients, the level of LIN28B, which is an RNA binding protein, decreases and tumor necrosis factor alpha (TNF-α) expression increases as a result.^[11]^ TNF-α has been shown to trigger a procoagulant mechanism affects fibrinogen level via tissue factor.^[12]^ It is also known that increased serum levels of TNF-α lead to a decrease in albumin level by decreasing albumin gene expression.^[13]^ Studies have shown that elevated serum TNF-α levels result in a decrease in hemoglobin level.^[14]^

Marinobufagenin (MBG), a steroid, is produced from the placenta and adrenocortex.^[15]^ In previous studies, it has been shown that serum levels of MBG increase before hypertension develops, it is involved in the pathophysiology of pre-eclampsia and it has been shown to be involved in capillary leakage.^[16,17]^ Development of capillary leakage causes albumin to diffuse into the extracellular fluid, resulting in a decrease in serum albumin levels.

Based on this evidence, it is logical to investigate HALP and FAR for the prediction of PE. In previous studies examining the relationship between HALP, FAR, and PE; the correlation of these scores with the diagnosis and severity of PE has been examined, but whether they can be used for prediction in early gestational weeks has not been investigated. The aim of our study was to investigate whether inflammation occurring during abnormal placentation changes HALP and FAR values and whether this change is a parameter that can be used in the prediction of PE.

## 2. Materials and methods

### 2.1. Study design

In this retrospective observational study the medical records of patients who gave birth between November 2022 and April 2024 at Etlik City Hospital, a tertiary care center, were examined. Patients between 18 to 40 years of age, who had a live birth and whose results were including Hb, Alb, Lym, Plt, Fib, creatinine (Cr), alanine transaminase (ALT), aspartate aminotransferase (AST) values from the blood tests required for the study at the first visit to the hospital in the hospital electronic registration system were included in the study. Patients with liver and kidney failure, patients diagnosed with diabetes and patients with any known autoimmune disease (antiphospholipid syndrome, etc) were excluded from the study. The electronic medical records of the patients were examined and age, parity, systolic, and diastolic BP values at the time of admission for delivery, and Hb, Alb, Lym, Plt, Fib, Cr, ALT, AST values in the first trimester were collected. The gestational week information was collected by analyzing the ultrasonography records performed on the day she gave her blood parameters in the first trimester. SP and NSP were diagnosed according to the criteria determined by American College of Obstetricians and Gynecologists. For the diagnosis of PE, systolic BP ≥ 140 mm Hg and/or diastolic BP ≥ 90 mm Hg in 2 measurements made 4 hours apart after the 20th week of pregnancy in a woman without previous hypertension and various multisystem disorders (Plt count 1,00,000 × 10^9^/L with or without proteinuria; liver enzymes must be elevated to twice the upper limit of normal concentration); unexplained severe persistent right upper quadrant or epigastric pain; renal insufficiency (serum Cr ≥ 1.1 mg/dL or doubling of serum Cr concentration in the absence of renal disease); pulmonary edema; or unexplained new-onset headache unresponsive to acetaminophen.^[3]^ Using these criteria, 84 pregnant women who were diagnosed with SP, 84 pregnant women who were evaluated as NSP and 84 healthy pregnant women (control) were included in the study.

Ethics committee permission was obtained from the ethics committee of Ankara Etlik City Hospital with the date June 5, 2024 and number AESH-BADEK-2024-516.

### 2.2. Sample collection and analysis

Venous blood samples were collected from the patients and healthy control subjects in the sitting position before noon after fasting for at least 8 hours. Blood samples were collected in 1 to 5 mL BD Vacutainer SST II Advance Blood Collection Tubes gel dry tube (lot 4024601; Becton Dickinson, Plymouth, UK) for serum and in a 3 mL BD Vacutainer™ Blood Collection K2EDTA tube (lot 4141891; Becton Dickinson, Plymouth, UK) for complete blood count. Serum was separated from the blood samples by centrifugation at 1500 g for 10 minutes after the tube with gel separator was left for 30 minutes. ALT, AST, Alb, Fib, and Cr levels were measured by electrochemiluminescence immunoassay using Cobas e801 (Roche Diagnostics, Mannheim, Germany). Complete blood count was performed by fluorescence flow cytometry method on a Sysmex XN-1000 (Sysmex Europe GmbH, Bornbarch 1, 22848 Norderstedt, Germany).

### 2.3. Statistical analysis

Age, parity, Hb, Alb, Lym, Plt, Fib, Cr, ALT, and AST values, systolic BP and diastolic BP measured at the time of hospitalization for delivery and the gestational week at which these tests were performed were recorded and analyzed with SPSS 25.0 (IBM Corp., Armonk). One-way ANOVA test was used for statistical analysis of the parameters analyzed receiver operating characteristic (ROC) curve and area under curve (AUC) were used to calculate sensitivity, specificity, positive predictive value (PPV) and negative predictive value (NPV) for the value correlated with PE severity. The number of patients to be included in the study was calculated a priori by power analysis. In the power analysis, the effect size for the analysis with 1-way ANOVA test was 0.25 (medium) with 95% power and the total number of patients required was 252.

## 3. Results

A total of 252 pregnant women were divided into 3 groups: healthy normotensive pregnant controls (control, n = 84), NSP (n = 84), and SP (n = 84). Table 1 shows the mean age, gestational week at the time of blood sampling, parity and systolic and diastolic BP values measured at hospitalization for delivery in the control, NSP and SP groups. There was a significant difference between the mean ages of the 3 groups (*P* < .01). The mean age of the SP group (32.20 ± 5.58) was significantly higher than that of the control group (28.52 ± 5.67; *P* = <.01), but there was no significant difference between the NSP (30.56 ± 5.58) and SP groups (*P* = .621; Table 1). The age difference between NSP and SP group was also not significant (*P* = .158). There was no significant difference in the mean gestational age at the time of blood sampling between the control, NSP, and SP groups (*P* = .856). There was no significant difference between the parity counts of the 3 groups (*P* = .815). The systolic BP value measured at hospitalization for labor was 114.21 ± 11.21 in the control group, 114.21 ± 11.21 in the NSP group and 159.00 ± 10.89 in the SP group and there was a significant difference between them (*P* < .01). Diastolic BP values also showed a significant difference between the control, NSP and SP groups (70.65 ± 8.09, 91.27 ± 5.81, 97.95 ± 6.54, *P* < .01, respectively; Table 1).

**Table 1:** Maternal characteristics of the groups.

	Control (n = 84)	NSP (n = 84)	SP (n = 84)	*P*-value	Control vs NSP *P*-value	Control vs SP *P*-value	NSP vs SP *P*-value
Age	28.52 ± 5.67	30.56 ± 5.58	32.20 ± 5.91	<.001	.061	<.001	.158
Gestational week of blood sampling	8.12 ± 1.23	8.26 ± 1.16	8.06 ± 1.33	.856	–	–	–
Parity	1.18 ± 0.26	1.24 ± 0.33	1.22 ± 0.29	.815	–	–	–
Systolic BP (mm Hg)	114.21 ± 11.21	144.19 ± 4.93	159.00 ± 10.89	<.001	<.001	<.001	<.001
Diastolic BP (mm Hg)	70.65 ± 8.09	91.27 ± 5.81	97.95 ± 6.54	<.001	<.001	<.001	<.001

BP = blood pressure, NSP = non-severe pre-eclampsia, SP = severe pre-eclampsia.

Table 2 shows the distribution of the parameters tested in our study according to the groups and the results of post hoc analyses of the values with significant differences between them. Hb, Lym, ALT, AST, Cr, Fib, and HALP values showed no significant difference between the groups (*P* values were .823, .089, .815, .384, .159, .171, .343, respectively; Table 2). Plt value showed a significant difference between the groups (*P* < .01; Table 2). In the post hoc analysis for Plt value, the value of the control group was significantly lower than the NSP group (respectively; 244.10 ± 63.80, 286.92 ± 73.58, *P* < .01; Table 2) and there was a significant difference between NSP and SP groups (respectively; 286.92 ± 73.58, 249.18 ± 74.0, *P* = .02; Table 2). Alb value was 36.29 ± 2.85 in the control group, 35.26 ± 3.91 in the NSP group and 34.06 ± 4.87 in the SP group (Table 2). In the post hoc analysis, there was a significant difference between the control group and the SP group (*P* = .001), while no significant difference was observed between the control and NSP group (*P* = .227) and between the NSP and SP groups (*P* = .129; Table 2). There is a significant difference between the 3 groups for FAR (*P* = .006). In the post hoc analysis, there was a significant difference between the control group (13.19 ± 2.86), NSP group (14.43 ± 2.93) and SP group (14.73 ± 3.75; *P* = .038, *P* = .007, respectively) and no significant difference between NSP and SP groups (Table 2).

**Table 2:** Distribution of tested values according to groups.

	Control (n = 84)	NSP (n = 84)	SP (n = 84)	*P*-value	Control vs NSP *P*-value	Control vs SP *P*-value	NSP vs SP *P*-value
Hemoglobin (g/dL)	11.57 ± 1.44	11.70 ± 1.31	11.67 ± 1.43	.823	–	–	–
Platelet (×10^3^/μL)	244.10 ± 63.80	286.92 ± 73.58	249.18 ± 74.01	<.001	<.001	.891	.002
Lymphocyte (×10^3^/μL)	1.78 ± 0.66	2.08 ± 0.96	2.00 ± 0.98	.089	–	–	–
Albumin (g/dL)	36.29 ± 2.85	35.26 ± 3.91	34.06 ± 4.87	.002	.227	.001	.129
AST (IU/L)	19.12 ± 7.27	18.45 ± 7.03	18.57 ± 6.88	.815	–	–	–
ALT (IU/L)	12.56 ± 6.73	14.15 ± 8.47	13.83 ± 7.86	.384	–	–	–
Creatinine (mg/dL)	0.52 ± 0.13	0.55 ± 0.13	0.55 ± 0.12	.159	–	–	–
Fibrinogen (mg/dL)	475.72 ± 93.07	506.41 ± 101.29	493.41 ± 116.17	.171	–	–	–
HALP score	3.23 ± 1.50	3.10 ± 1.37	3.48 ± 2.14	.343	–	–	–
FAR	13.19 ± 2.86	14.43 ± 2.93	14.73 ± 3.75	.006	.038	.007	.828

ALT = alanine transaminase, AST = aspartate aminotransferase, FAR = fibrinogen/albumin ratio, HALP = hemoglobin, albumin, lymphocyte, and platelets’, NSP = non-severe pre-eclampsia, SP = severe pre-eclampsia.

**Figure 1.**
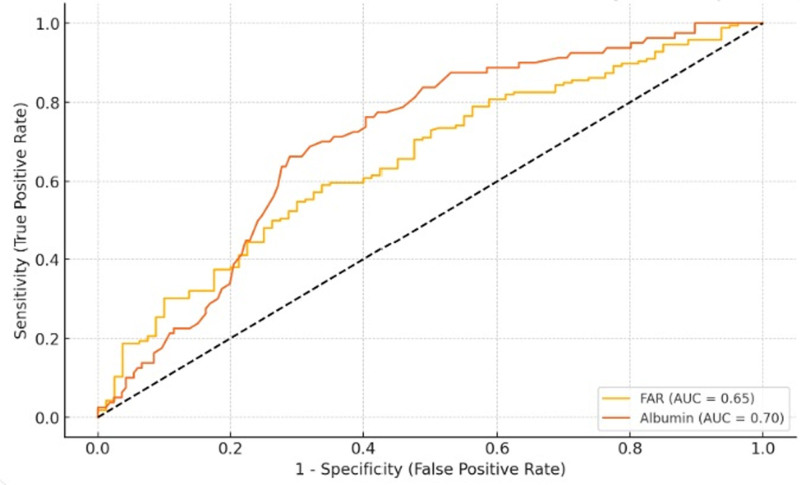
Receiver operating characteristic curve for fibrinogen/albumin ratio and albumin. AUC = area under curve, FAR = fibrinogen/albumin ratio.

Table 3 and Figure 1 shows the ROC curve results for FAR and Alb variables. The calculated AUC for the FAR variable was 0.650 (95% CI: 0.576–0.726), sensitivity 0.59, specificity 0.66, PPV 0.784, NPV 0.438, and the best cutoff value was calculated as 13.89 (*P* < .001; Table 3 and Fig. 1). For the Alb variable, AUC 0.703 (95% CI: 0.638–0.765), sensitivity 0.66, specificity 0.71, PPV 0.52, NPV 0.81, and the best cutoff value was calculated as 35.9 (*P* < .001; Table 3 and Fig. 1).

**Table 3:** Receiver operating characteristic curve results for fibrinogen/albumin ratio and albumin variables.

		Value	*P*-value	Cutoff value	%95 Confidence interval	
					Lower limit	Upper limit
FAR	AUC	0.650	<.001	13.89	0.576	0.726
	Sensitivity	0.59				
	Specificity	0.66				
	Positive predictive value	0.784				
	Negative predictive value	0.438				
Albumin	AUC	0.703	<.001	35.9	0.638	0.765
	Sensitivity	0.66				
	Specificity	0.71				
	Positive predictive value	0.52				
	Negative predictive value	0.81				

AUC = area under curve, FAR = fibrinogen/albumin ratio.

## 4. Discussion

In this study, the potential role of HALP score and FAR values measured in the first trimester in the prediction of PE was investigated. The results showed that the FAR value was significantly higher in patients with NSP and CP compared to healthy pregnant women. For HALP score, there was no significant difference between the patients in NSP and SP groups and the control group. Alb value showed a significant difference between the control and SP groups, while no significant difference was observed between the control and NSP and NSP and SP groups.

The results of the HALP score showing no significant difference between the NSP and SP groups and the control group suggest that the HALP score may have limited utility in the early diagnosis of PE. Previous studies suggest that the HALP score may be used as a prognostic indicator in various inflammatory and oncological conditions.^[8]^ When the literature is examined, there are limited studies that investigated the relationship between HALP score and PE. Previously, Dal et al showed that the HALP score was lower in pregnant women with PE compared with normotensive pregnant women in a study conducted in 2024, but in this study, the HALP score was examined at the gestational weeks when PE was diagnosed.^[9]^ In our study, the first trimester HALP score was compared between the control and PE groups and no significant difference was found between them. The difference between the results of Dal et al and our findings suggests that HALP score may be valuable in the diagnosis of PE but is not sufficient in the prediction of PE. We think that more research is needed for the use of HALP score in the diagnosis and prediction of PE.

It was observed that the FAR value increased significantly with PE severity from the control group to the SP group. We think that this increase in FAR values is due to the increase in Fib levels as a result of the procoagulant system being activated by TNF-α, and the decrease in albumin levels due to capillary leakage resulting from both the suppression of gene expression and the increase in MBG.^[12,13,17]^ In addition, FAR elevation is compatible with the study by Ren et al and shows that FAR elevation is increased not only when PE occurs but also before PE.^[10]^

The ROC analysis results of our study revealed that FAR had a sensitivity of 0.59 and a specificity of 0.66 in predicting PE. However, the area under the ROC curve was at intermediate levels (0.65), suggesting that FAR alone is not a powerful diagnostic tool, but may contribute to the prediction of PE when used in combination with other biochemical parameters. In our study, AUC values were found to be 0.65 for FAR and 0.70 for albumin. These AUC values indicate only moderate predictive accuracy and, while they fall short of current standard angiogenic markers such as the sFlt-1/PlGF ratio, which typically have AUC levels above 0.85 and high diagnostic accuracy, they offer advantages in terms of being widely available in laboratories, low cost, and not requiring specialized equipment. In primary care settings where access to advanced diagnostic tests is limited, markers such as FAR and albumin can be used for risk stratification and may help identify patients who require further testing. Additionally, the use of these parameters in combination with existing biomarkers in multivariate models may enhance overall predictive power. Our hypothesis is that evaluation of FAR together with biomarkers such as PlGF and sFlt-1, which are frequently investigated in the literature, may provide a more comprehensive approach in the prediction of PE.^[6]^ We think that randomized controlled studies should be performed to investigate the relationship between the use of FAR, PlGF and sFlt-1 together and the improvement in the prediction of PE.

According to our results, the AUC value of Alb in ROC analyses was 0.70, the sensitivity rate was 66% and the specificity rate was 71%, indicating that Alb provides a moderate accuracy in the diagnosis of PE. Alb is a negative acute phase reactant that decreases during inflammation, and decreased levels in PE patients may be considered as a result of increased TNF-α levels. Studies in the literature have also shown that Alb is associated with systemic inflammation and endothelial dysfunction involved in the pathophysiology of PE. For example, studies by Redman et al and Chaiworapongsa et al revealed that low Alb levels may be associated with the development of PE and may reflect the severity of the disease.^[18]^ To increase the reliability of albumin levels, we included people whose blood samples were taken in the morning after at least 8 hours of fasting and who had no liver disease, but the diet and nutritional status of individuals may also affect albumin levels. However, it is remarkable that it is a marker with moderate power to be used alone for prediction; therefore, we think that more studies are needed for its use in combination with other markers.

In our study, early-onset and late-onset PE were also not analyzed by subgroup analysis. Considering the different pathophysiological pathways, we think that the power of FAR and albumin to discriminate early-onset and late-onset PE in the prediction of PE should be evaluated in future studies.

Since our study was retrospective and had a limited sample group and did not evaluate early and late-onset PE separately, our results regarding the prediction of PE by HALP score and FAR are limited. Therefore, we think that our results should be supported by more comprehensive randomized controlled studies.

## 5. Conclusions

In conclusion, in this study, it was observed that FAR and Alb may be a useful parameter in the prediction of PE, but HALP score may play a limited role. In future studies, it is recommended that new parameters should be included in order to make PE prediction more accurately with larger sample groups and different biomarker combinations.

## Author contributions

**Conceptualization:** Murat Polat, Aziz Kindan.

**Data curation:** Mehmet Alican Sapmaz, Sait Erbey, Muhammed Said Ruzgar.

**Formal analysis:** Murat Polat, Mehmet Alican Sapmaz.

**Investigation:** Murat Polat, Mehmet Alican Sapmaz, Sait Erbey, Muhammed Said Ruzgar, Aziz Kindan.

**Methodology:** Murat Polat, Aziz Kindan.

**Project administration:** Murat Polat.

**Resources:** Murat Polat, Sait Erbey, Muhammed Said Ruzgar.

**Software:** Mehmet Alican Sapmaz.

**Supervision:** Aziz Kindan, Inci Kahyaoglu.

**Validation:** Sait Erbey, Aziz Kindan.

**Visualization:** Sait Erbey.

**Writing** – **original draft:** Murat Polat, Inci Kahyaoglu.

**Writing** – **review & editing:** Inci Kahyaoglu.
